# CohortExplorer: A Generic Application Programming Interface for Entity Attribute Value Database Schemas

**DOI:** 10.2196/medinform.3339

**Published:** 2014-12-01

**Authors:** Abhishek Dixit, Richard J B Dobson

**Affiliations:** ^1^Institute of PsychiatryNIHR Biomedical Research Centre for Mental Health & Biomedical Research Unit for DementiaSouth London and Maudsley NHS Foundation Trust & Institute of Psychiatry, Kings College LondonLondonUnited Kingdom

**Keywords:** entity-attribute-value schema, biobank database, clinical information systems, CDISC ODM, SQL

## Abstract

**Background:**

Most electronic data capture (EDC) and electronic data management (EDM) systems developed to collect and store clinical data from participants recruited into studies are based on generic entity-attribute-value (EAV) database schemas which enable rapid and flexible deployment in a range of study designs. The drawback to such schemas is that they are cumbersome to query with structured query language (SQL). The problem increases when researchers involved in multiple studies use multiple electronic data capture and management systems each with variation on the EAV schema.

**Objective:**

The aim of this study is to develop a generic application which allows easy and rapid exploration of data and metadata stored under EAV schemas that are organized into a survey format (questionnaires/events, questions, values), in other words, the Clinical Data Interchange Standards Consortium (CDISC) Observational Data Model (ODM).

**Methods:**

CohortExplorer is written in Perl programming language and uses the concept of SQL abstract which allows the SQL query to be treated like a hash (key-value pairs).

**Results:**

We have developed a tool, CohortExplorer, which once configured for a EAV system will "plug-n-play" with EAV schemas, enabling the easy construction of complex queries through an abstracted interface. To demonstrate the utility of the CohortExplorer system, we show how it can be used with the popular EAV based frameworks; Opal (OBiBa) and REDCap.

**Conclusions:**

The application is available under a GPL-3+ license at the CPAN website. Currently the application only provides datasource application programming interfaces (APIs) for Opal and REDCap. In the future the application will be available with datasource APIs for all major electronic data capture and management systems such as OpenClinica and LabKey. At present the application is only compatible with EAV systems where the metadata is organized into surveys, questionnaires and events. Further work is needed to make the application compatible with EAV schemas where the metadata is organized into hierarchies such as Informatics for Integrating Biology & the Bedside (i2b2). A video tutorial demonstrating the application setup, datasource configuration, and search features is available on YouTube. The application source code is available at the GitHub website and the users are encouraged to suggest new features and contribute to the development of APIs for new EAV systems.

## Introduction

Electronic data capture (EDC) and electronic data management (EDM) systems are a key requirement for studies in modern biomedical science. Such systems are developed to centrally manage the recruitment and storage of participant details. They typically comprise a powerful database engine accessible over the network using Web-based technologies. Such systems include built-in sanity checking and quality control procedures to ensure the data captured is consistent and well formatted for ease of downstream analysis. Some of the popular EDC and EDM systems include OpenClinica, LabKey, Onyx, Opal, REDCap, entity-attribute-value **(**EAV) with classes and relationships (EAV/CR), and Informatics for Integrating Biology & the Bedside (i2b2 ) [1-7].

Typically, EDC and EDM systems employ a generic EAV schema. Through the use of such a schema, hundreds of clinical attributes (or variables) can be stored in a single table without having to create multiple tables. Additionally, more attributes can be easily added without changing the underlying schema [8-11]. The EAV model can be viewed as a database table with three columns; one column specifies the entity (eg, participant ID), one for the attribute (eg, cognitive test), and one for the value of the attribute (eg, cognitive test score) [12]. If the study is longitudinal with multiple follow-up visits then an additional column is often used to store the visit number. In longitudinal studies, the combination of entity_id and visit number can act as a primary key (a composite primary key).

Although the EAV model provides a great deal of flexibility in storing data, such a schema requires the use of complex structured query language (SQL) to extract subsets of data from the tables [13-16]. In addition, the choice of the system depends on the study requirements. This poses a problem for the researchers as different EDC and EDM systems differ in their graphical user interface, data model and sometimes the vendor/relational database management system (eg, OpenClinica can be implemented in Oracle, PostgreSQL, Labkey in PostgreSQL, REDCap, and Opal in MySQL) thereby increasing the burden of understanding and using the underlined data model before querying datasources.

To address this problem we have developed CohortExplorer, a generic framework that allows the detailed exploration of clinical data stored under the EAV schema which is organized into a survey format (questionnaires/events, questions, and values) using a standard search interface. The main objectives were to: (1) standardize the interface to EAV databases; (2) enable user-friendly querying of entities and variables (ie, meta data) at depth; and (3) provide the functionality to export the data which can be readily parsed and loaded by statistical software such as R for downstream analysis [17]. CohortExplorer has no schema and solely depends on the datasource API (discussed in the next section) and read-only connection made to the clinical repository implementing the EAV schema.

By way of example, we demonstrate the utility of CohortExplorer by connecting to and querying two commonly used EDC and EDM systems, namely Opal [4] and REDCap [5] both implementing their own version of the EAV schema in MySQL. Opal and REDCap greatly vary in their functionality; Opal is developed to manage the participants (ie, EDM) recruited as part of the clinical studies and relies on Onyx [3], its sister software to recruit the participants (ie, EDC). Both Onyx and Opal are developed as a part of OBiBa [18], a core project of the Population Project in Genomics Consortium (P3G), committed towards building high quality open source systems for biobanks. All OBiBa software along with their source code is available under the open source GPL3 license. REDCap encompasses both participant recruitment and management functionalities. REDCap was developed at the Vanderbilt University and is currently comprised of over 900 active institutional partners with bases all over the world. REDCap, unlike systems developed by OBiBa, is not open source but is available at no charge.

## Methods

### CohortExplorer Core Components and Implementation

CohortExplorer has three main components: (1) a datasource API and configuration file; (2) an SQL abstraction layer; and (3) a command line search interface. Both the SQL abstraction layer and command line query interface have been implemented using object oriented Perl [19] programming language. Data captured by the systems (questionnaires, surveys, and forms) are referred to herein as tables and the questions, which form part of the study, are termed variables with values being the answers to the questions.

First, the easy part of building an EAV-schema-agnostic API is achieving backend independence. CohortExplorer implements backend independence by the use of Perl module DBI [20]. DBI is independent of any database available in the backend and is responsible for taking all SQL commands and dispatching them to the appropriate driver for execution. Using CohortExplorer's datasource API (a Perl class) the users can define the entity, table, and variable structure under the EAV system. By structure we mean what database tables and columns are to be consulted to query data and meta data. The organization of entities, tables, and variables can be transformed into Perl hash (ie, data structure with key value pairs) using SQL::Abstract [21] discussed below. The Perl hash for entity, table and variable can vary with variation in EAV schema. In addition, the user authentication mechanism can also be defined in the datasource API. The datasource configuration file allows the user to define datasource settings including database connection details like dsn, username, and password (ie, it is the connector). The documentation detailing the API is available online [22]. A video tutorial aiming to give users an insight into application set-up including datasource configuration is also available online [23]. The tutorial with examples demonstrates various search features offered by the application.

**Figure 1:**
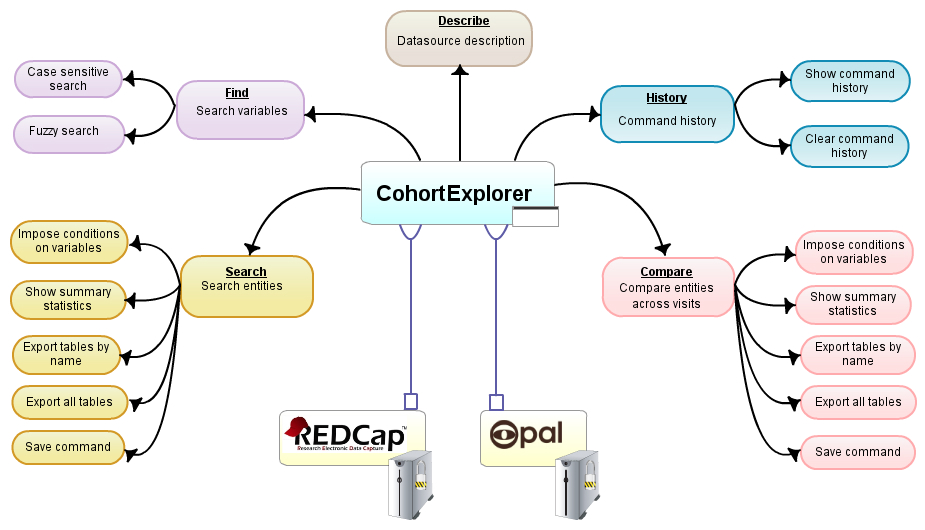
Using the datasource API, CohortExplorer, via the authentication mechanism, is able to connect to secure EAV clinical repositories such as REDCap and Opal. The connection made to the clinical repository is expected to be read-only. Once connected, the user can explore the datasources stored within the repositories using the abstracted command line search interface.

Currently, CohortExplorer comes with built-in APIs for Opal and REDCap each catering to their own authentication mechanism and variation in the EAV schema. Therefore, the datasources stored within these systems can be queried using the current set-up (Figure 1).

We intend to provide APIs for other EDC and EDM systems such as LabKey [1] and OpenClinica [2] so the users can query the repositories implemented using these systems with same ease as Opal and REDCap. Opal and REDCap were the starting point considering their use at our institution. The application source code is available on GitHub, a popular platform for sharing and developing code [24]. The user community is encouraged to contribute to the development of APIs appertaining to new EAV systems.

The security in CohortExplorer is implemented using the built-in authentication mechanism, setuid and Linux file permissions. The application runs under the taint mode which sets up special security checks including the check for unauthorized input. The security features ensure the user running the application has no access to the configuration files containing the connection details of the clinical repositories, the administrator is expected to create a read-only connection to the repository. Moreover, the application can be easily made to pay attention to user permission assigned within the repository. For example, the REDCap datasource API ensures only users who are allowed to export data in REDCap can use CohortExplorer. The API takes into account what variables and records are accessible to the user within REDCap. If some user is prohibited from viewing the identifiable information on participants in REDCap the API makes sure the user does not have access to the variables pertaining to the participant identifiable information (eg, participant's name, address, etc).

Second**,** at its core, CohortExplorer is powered by the SQL abstraction layer implemented using the Perl module, SQL::Abstract [21]. The abstraction layer serves two main purposes^.^ Firstly, it allows SQL statements to be treated as a hash with SQL components (ie, -columns, -from, -where, -group_by, -order_by, and -having) as keys in the hash. The SQL statements to query data and meta data can easily be constructed from the entity, table and variable structures defined in the datasource subclass. As the EAV datasource can be cross-sectional or longitudinal, the second feature of the abstraction layer is that it enables the SQL generating engine to generalize the EAV schema as a 1 or 2 table database (static or dynamic) depending on the datasource type, hence making the easy and flexible construction of complex and dynamic SQL statements with placeholders. This is done to address the data heterogeneity at the forms/questionnaires/surveys level. The forms, which are only used once throughout the study, are grouped under static table (eg, participant demographics). This table is created by grouping or aggregating the form data on entity_id. Such table is applicable to cross-sectional studies but may also apply to longitudinal datasources. The forms which are used repeatedly throughout the study in the form of follow-up visits are grouped under dynamic table (eg, cognitive assessments). The dynamic table is created by grouping the data on the concerned forms on the entity_id and the visit number. Currently the application does not support querying datasources with multiple arms. In future the application may consider other table structures to address variation in data with respect to arms.

**Figure 2:**
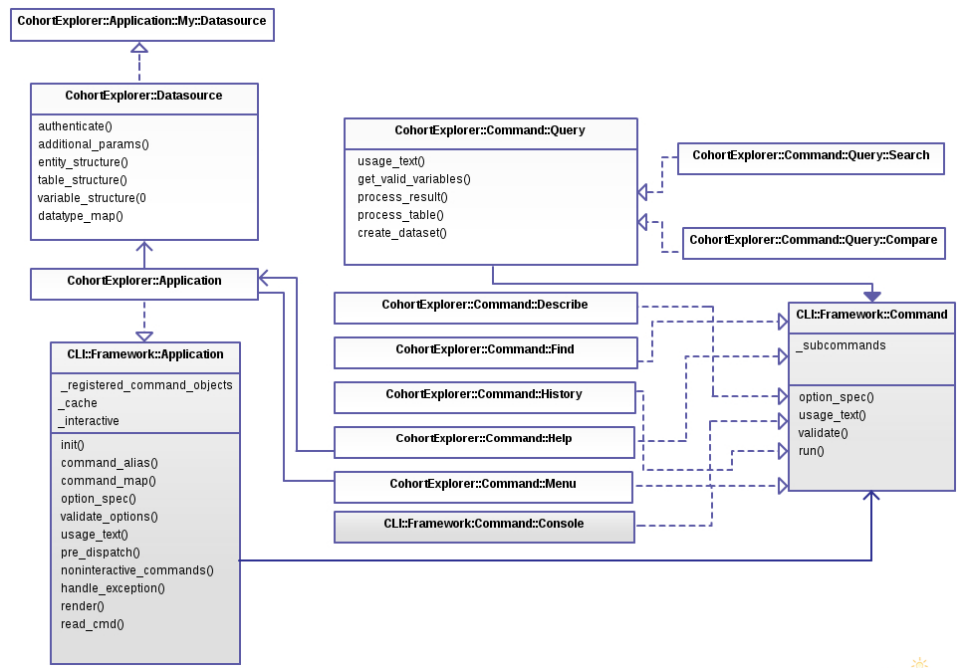
The figure shows the classes implemented under CohortExplorer. CohortExplorer::Application is inherited from CLI::Framework::Application (super class) and overrides the methods shown. All command classes are inherited from CLI::Framework::Command which is an abstract factory (dotted arrows). The meta commands such as menu and help are application aware commands with direct access to the application object. The application class dispatches the command objects. CohortExplorer::Command::Query extends CLI::Framework::Command (dark head arrow) and acts as an interface to search and compare command classes. CohortExplorer::Application class uses CohortExplorer::Datasource (an abstract class) to initialize the datasource. CohortExplorer::Datasource provides hooks or methods (also shown) which are implemented by subclasses such as CohortExplorer::Application::My::Datasource corresponding to electronic data capture and management systems such as Opal and REDCap.

**Figure 3:**
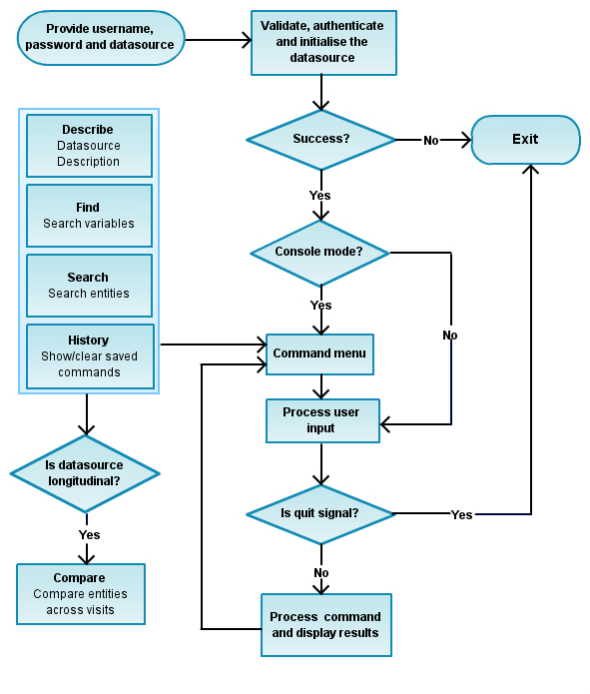
The flow chart showing the overall work flow of CohortExplorer. The user runs CohortExplorer by providing the datasource name, username, password and the command to execute. When running the application in console/interactive mode (determined by the console command) a menu of available commands is displayed. The user inputs the command along with command options and arguments (if applicable), the application processes the command and displays the results. When running the application on standard Linux shell the application simply processes the command input, displays the results and terminates the connection.

Third, the command line interface (CLI) is implemented using the Perl module CLI::Framework [25] (See Figure 2) and enables the user to query the clinical datasources. The CLI has two main components: (1) Application - this component authenticates the user and initializes CohortExplorer for the user specified datasource and dispatches the supplied command for further processing (See Figure 3); (2) Command - this component does the command specific processing and returns the output to the application component for display. The command component is divided into 5 main commands each of which performs a specific operation as described in Textbox 1.

Each command has a mandatory help section which details command usage with examples. CohortExplorer can also be run on the standard Linux shell so the user can easily set-up a report scheduling using the Linux in built Cron functionality.

**Command components.**

1. **describe** - This command prints the datasource description in a tabular format where the table header is the entity count (ie, number of participants in the datasource/study) and table body contains the information appertaining to each table in the datasource. The first column in the table body is the table name (ie, questionnaire/surveys/forms) followed by table attributes (eg, variable count, associated label, etc) specified in the datasource API.
2. **find** - This command allows the user to find recorded variables using keywords which can be utilized to build an entity search query. The user can perform both case insensitive and fuzzy searches. The command prints the variable dictionary (ie, meta data) of variables meeting the search criteria in a tabular format where the first column is the name of the variable, second column is the table which records the variable and other columns include variable attributes (eg, variable type, categories, associated label, etc) specified in datasource API. The command looks for the presence of keywords in all variable attributes.
3. **search** - This command allows the user to search entities using variables of interest. The user can also impose conditions on variables using all valid SQL operators; =, !=, >, <, >=, <=, in, not_in, like, not_like, ilike, between, not_between, regexp, and not_regexp. The command includes auto-completion enabling the user to enter the first few characters of some command option/argument (eg, export directory, variable or table name) and press the completion key (ie, TAB) to fill-in the rest of the characters. At any time in CohortExplorer's console/interactive mode the user is able to view all tables and variables they have access to by simply pressing the TAB key. In addition, the command allows the user to view descriptive statistics and export data in csv format which can be easily parsed in statistical software like R for downstream analysis. The search command is available to both cross sectional and longitudinal datasources. When calculating descriptive statistics for variables belonging to dynamic tables the command groups the variables by visit. The command also includes a bookmarking feature which allows the user to save commands for future use.
4. **compare -** As the name suggests, the compare command allows the user to compare entities across visits. The command is only available to longitudinal datasources. The command allows the user to search and impose conditions at a visit level. Prefixes vAny, vLast, v1, v2, etc are added to variable names. For example: v1.var represents first visit of the variable 'var', v2.var represents second visit, vAny.var implies any visit, vLast.var last visit, and 'var' in this command simply represents all visits. The prefix vAny and vLast are abstract terms as vAny and vLast can be any visit (generally the last time a variable was recorded for some entity is not known in advance so practically any visit can be the last visit). The data exported via this command is formatted horizontally (ie, repeating variables) unlike the search command which exports the data vertically (ie, repeating entities) where each row represents an entity followed by the user provided visit variables (ie, dynamic table) or simply variables in case of static tables. The statistics produced in this command are calculated with respect to the entity_id and the number of observations for each variable is equivalent to the number of times or visits each variable was recorded for each entity.
5. **history -** The user can keep track of their previously saved commands using the history command. By specifying the show option the user can view all their saved commands along with the date-time stamp. The user can re-run any of the previously saved command or use the information in the commands (ie, options arguments) to build new commands.

## Results

### Case Studies

#### Overview

To evaluate the utility and feasibility of CohortExplorer we connected the system to two real-world datasources, an Alzheimer's and Dementia biomarker study and the National Institute for Health Research **(**NIHR) BioResource for Mental Health based at the Institute of Psychiatry, Kings College London, United Kingdom.

The NIHR Alzheimer's and Dementia datasource is powered by Opal (OBiBa) [4] and stores the data from two cohorts; namely AddNeuroMed (European Union funded European Middleware Initiative) [26-27] and Kings Health Partners - Dementia Case Register (DCR) [28-29]. The data comprises participant and informant interviews conducted using Onyx (OBiBa) [3] longitudinally. The Onyx interview is comprised of7 main questionnaire categories: consent, demographics, physical measurement and samples obtained, disease history, family history, cognitive tests, and diagnosis.

Data collected by The NIHR BioResource for Mental Health [30] is stored in REDCap. This longitudinal study aims to collect 50,000 samples over the next 5 years from patients registered with South London And Maudsley NHS Foundation Trust (SLAM) and King's Health Partners. The project collects data on patients' demographics (eg, age, sex, ethnicity, etc) along with blood and saliva samples for molecular analysis, which includes developing new diagnosis tests, identifying new drug targets, and understanding the causes of different mental disorders.

Below, we provide examples of distributed queries that can easily be performed on the two clinical datasources using CohortExplorer's Opal and REDCap datasource API.

#### Alzheimer's and Dementia Datasource (Opal)

**Figure 4:**
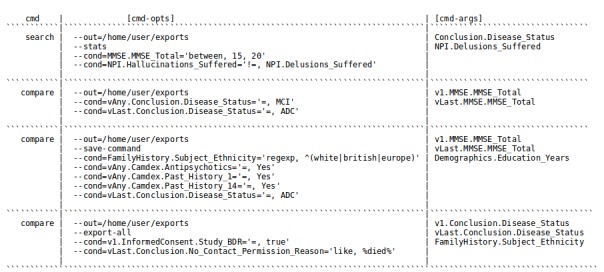
CohortExplorer commands for the Alzheimer's and Dementia datasource. For better understanding the commands are divided into their respective components namely; command name, command options and command arguments.

Questions we can answer using CohortExplorer's Opal datasource API (Figure 4): (1) during the course of the study how many participants with Mini Mental State Examination (MMSE) scores between 15 and 20 have had a history of hallucinations but not delusions or vice versa? We would like to know their disease status; (2) how many participants who previously had mild cognitive impairment have been diagnosed with Alzheimer's disease? We would also like to see their MMSE total at first and last visit; (3) at any visit during the study how many non-European females receiving anti-psychotic medication have been diagnosed with Alzheimer's disease? We would also like to know their MMSE scores and if they had ever suffered with high blood pressure and diabetes; and (4) how many participants have consented for brains for dementia research study? For all consented participants export complete data and show ethnicity, disease status at first and last visit.

#### NIHR BioResource Datasource (REDCap)

**Figure 5:**
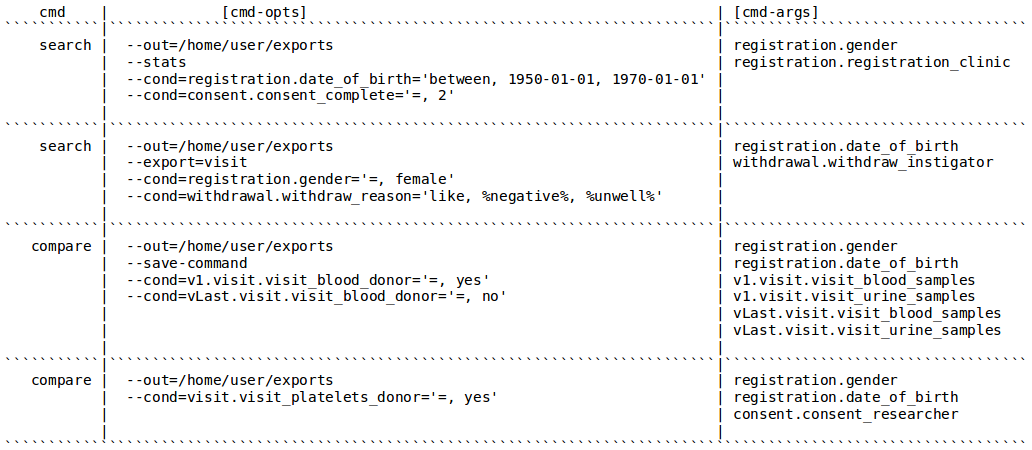
CohortExplorer commands for the NIHR BioResource for Mental Health datasource.

Questions we can answer using CohortExplorer's REDCap datasource API (Figure 5): (1) how many participants in the study were born between 1950 and 1970? For all consented participants, produce summary statistics showing percentage breakdown by gender and registration clinic; (2) how many females have withdrawn from the study citing negative media reports and health reasons? Show date of birth of all females; (3) how many participants have donated blood on their first visit but not the last visit? For all participants meeting the query criteria obtain data on gender, date of birth, and samples collected; and (4) how many participants have donated blood platelets in all visits? For all resulting participants show gender, date of birth, and the investigator who took the consent.

## Discussion

### Principal Findings

CohortExplorer provides a secure and standard platform with which to query clinical repositories that are based on the EAV framework such as Opal and REDCap. The application relies on a read only database connection to the repository (via the datasource configuration file and datasource API). For longitudinal studies, CohortExplorer provides summary statistics both at the visit level as well as at the entity level. Moreover, the output from the application can be easily parsed by statistical software such as R for downstream analysis and the commands can be bookmarked for future use. The application runs on standard Linux shell thus making scheduled reports possible through the cron daemon. The application also supports the auto-complete or tab completion functionality making it easier for the user to provide variables and table names. The functionality can be helpful considering clinical variables can have long names.

CohortExplorer provided basic authorization and pays attention to the user permissions as implemented by the parent repository.

One of the main advantages of CohortExplorer is that the search interface is independent of the system storing the clinical data. This feature is of particular importance considering most of the EDC and EDM systems differ significantly in their query interface and researchers involved in multiple studies end up using multiple systems based on the study requirements. Deployment of CohortExplorer will lower the burden on researchers and data managers to learn and use the underlining data model before querying for entities of interest. With minimal training the researchers and data managers can use CohortExplorer to generate hypotheses, reports, and also to test the data accuracy.

CohortExplorer is written in Perl with CLI::Framework and SQL::Abstract as main modules. The application can be installed with all of its dependencies and the user manual via its Debian package which is available online [31]. As the application implements SQL abstraction it is compatible with other relational database management systems such as Oracle, Microsoft SQL Server, and PostgreSQL. However, this feature is yet to be tested. The Debian package includes Opal and REDCap APIs. The user is encouraged to use these as examples when trying to create a datasource API for a new EAV schema. The application is supported by active development and users are encouraged to suggest new features and get involved in development on GitHub [24]. At present, the application is only compatible with EAV systems that fit into a survey format (questionnaires/events, questions, and values) in other words, the CDISC Observational Data Model (ODM). Further work is needed to make the application compatible with EAV schemas where the metadata is organized into hierarchies such as i2b2.

The future work also includes extending the application to EDC and EDM systems implemented in Oracle, PostgreSQL, and Microsoft SQL server such as LabKey and OpenClinica.

### Conclusions

CohortExplorer provides a user-friendly and generic approach to slice and dice clinical datasources stored under the EAV format. For biomedical researchers, CohortExplorer provides an easy to understand view of the unstructured and complex clinical data. The application is available as open source under the GPL-3+ license. The source-code, Debian package and manual are available online [24,31]. A video tutorial demonstrating the application set-up and features is also available online [23]. The tutorial aims to give users an insight into the application set-up, datasource configuration, and query features.

## Multimedia Appendix 1

CohortExplorer source code, documentation and the Debian package.

## Abbreviations

API: application programming interface
CDISC: Clinical Data Interchange Standards Consortium
EAV: entity-attribute-value
EDC: electronic data capture
EDM: electronic data management
i2b2: Informatics for Integrating Biology & the Bedside
NIHR: National Institute for Health Research
ODM: observational data model
SLAM: South London and Maudsley NHS Foundation Trust
SQL: structured query language
